# The difference between patients with nephrotic syndrome and nephrotic-range proteinuria in IgA nephropathy: a propensity score matched cohort study

**DOI:** 10.1186/s12882-022-02799-3

**Published:** 2022-04-29

**Authors:** Hongfen Li, Fanghao Wang, Junya Jia, Tiekun Yan, Youxia Liu, Shan Lin

**Affiliations:** Department of Nephrology, Tianjin Medical University General Hospital, No. 154, Anshan Road, Heping District, Tianjin, China

**Keywords:** IgA nephropathy, Nephrotic syndrome, Nephrotic-range proteinuria, Propensity score matching

## Abstract

**Objective:**

To date, nephrotic syndrome (NS) has not been well characterized in patients with IgA nephropathy (IgAN). Whether decline in serum albumin is an ominous sign in IgAN patients with massive proteinuria remains unknown. In this study, we evaluated clinical and pathological features of IgAN with NS and compared the differences for these features and long-term outcomes between patients with nephrotic syndrome and nephrotic-range proteinuria.

**Methods:**

A retrospective study was conducted, enrolling 1013 patients with biopsy-proven IgAN. The primary endpoint was the composite of a doubling of the base-line serum creatinine, 50% reduction in eGFR, ESKD (eGFR < 15 ml/min per 1.73 m^2^) or death.

**Results:**

A total of 59 patients were presented with NS (5.8%). The patients with NS showed lower levels of hemoglobin, albumin and higher levels of serum creatinine, serum uric acid and urinary protein than patients without NS. As for pathological parameters, more patients with NS showed a higher prevalence of E1 lesions, T1/2 and C1/2 lesions. Furthermore, we used the propensity score matching method to select 57 patients with nephrotic-range proteinuria and normal serum albumin (NR group) who were comparable to 59 patients with NS. Patients with NS had lower levels of hemoglobin, albumin and IgG and higher levels of TC, LDL, FIB and D-dimer as well as more severe E1 and C1/2 lesions than those in NR group. The S1 lesion was more severe in the NR group than that in the NS group. There was no significant difference in long-term outcome between the two groups. In addition, we found that serum albumin level or the presence of hypoalbuminemia was not a risk factor affecting long-term outcome in patients with massive proteinuria.

**Conclusions:**

A prevalence of 5.8% of NS was presented in IgAN adult patients in our study. IgAN with NS patients had low levels of hemoglobin, albumin, high levels of serum creatinine, serum uric acid, urinary protein and more acute lesions. The prognosis of NS in patients with IgAN was not inferior to that of patients with nephrotic range proteinuria and normal serum albumin.

## Introduction

IgA nephropathy (IgAN) is one of the most common primary glomerular diseases in the world [1, 2]. The disease typically follows a slow but relentless clinical course that will develop end-stage renal disease (ESRD) in 30–40% of patients within 20 years after the diagnosis [1, 3]. The typical clinical manifestations of IgAN are hematuria and/or proteinuria [4]. IgAN patients with nephrotic syndrome (NS) are rare and account for about 5–10% [5–7]. To date, NS has not been well characterized in patients with IgAN. Although hypoalbuminemia is a fundamental characteristic of NS, there are some patients that do not develop hypoalbuminemia despite with massive proteinuria [8, 9]. Few studies focused on patients with nephrotic-range proteinuria and normal serum albumin. Whether decline in serum albumin is an ominous sign in IgAN patients with massive proteinuria remains unknown.

Therefore, in the present study, we intend to evaluate clinical and pathological features of IgAN with NS. And the differences for these features and long-term outcomes between patients with nephrotic syndrome and nephrotic-range proteinuria were also compared by propensity score matching method.

## Methods

### Study design and population

This was a retrospective study involving a cohort of IgAN patients at the Tianjin Medical University General Hospital. A total of 1013 patients with biopsy-proven IgAN from May 2010 to April 2021 were analyzed. Patients with secondary IgAN such as systemic lupus erythematosus and Henoch Schonlein purpura were excluded. IgAN with minimal change disease (MCD, defined as diffuse foot process fusion under electron microscope and mild mesangial hypercellularity) was also excluded. This study was approved by the ethics committee of the institute and all patients gave informed consent.

There are 59 patients with nephrotic syndrome (IgAN-NS group). We selected 57 patients with nephrotic-range proteinuria and normal serum albumin (IgAN-NR group) who were comparable to patients with IgAN-NS group by the propensity score matching method. The propensity score matching was conducted by 1:1 matching gender, age, blood pressure, renal function and proteinuria. Clinical demographics, estimated glomerular filtration rate (eGFR), systolic blood pressure (SBP), total cholesterol (TC), Low density lipoprotein (LDL), Fibrinogen (FIB), D-dimer (D-D) and proteinuria at the time of biopsy were recorded.

### Definitions

The nephrotic syndrome is defined as the presence of massive proteinuria (> 3.5 g/d) and hypoalbuminemia (less than 30 g/L). The definition of nephrotic-range proteinuria is massive proteinuria (> 3.5 g/d) but without hypoalbuminemia. The eGFR was calculated using the Chronic Kidney Disease Epidemiology Collaboration (CKD-EPI) equation [10]. The histological lesions were classified according to Oxford classification scores (MEST-C, M: mesangial hypercellularity; E: endocapillary hypercellularity; S: segmental glomerulosclerosis; T: tubular atrophy/interstitial fibrosis, and C: crescent) [11]. Immunosuppressive therapy was defined as treatment with steroids plus immunosuppressive agents, including cyclophosphamide, cyclosporine, or mycophenolate mofetil after kidney biopsy. A renin-angiotensin system inhibitor (RASI) was defined as the use of an angiotensin-converting enzyme inhibitor (AECI) and/or angiotensin receptor blocker (ARB) after biopsy. The primary endpoint was the composite of a doubling of the base-line serum creatinine, 50% reduction in eGFR, ESKD (eGFR < 15 ml/min per 1.73 m^2^) or death.

### Statistical analysis

Normally distributed continuous variables were expressed by mean ± standard deviation (SD) and compared using Student’s t test. Non-normally distributed continuous data were expressed as medians and interquartile ranges and compared using the Mann–Whitney U test. Dichotomous data were presented as numbers and percentages and compared using the χ 2 inspection. The predictive value for survival from a combined event was constructed by Kaplan–Meier survival curves and analyzed by a log-rank test. The odds ratio (OR) and the 95% CI between albumin and primary outcome was examined in logistic models. A two-tailed *P* < 0.05 was statistically significant. The analysis was performed using SPSS 25.0 software.

## Results

### Clinical and pathological characteristics of patients with nephrotic syndrome

A total of 1013 patients were enrolled in this study, including 59 patients with nephrotic syndrome (5.8%) and 954 (94.2%) patients without nephrotic syndrome (non-NS). As shown in Table 1, the patients with NS were older (42.69 ± 14.96 years vs. 37.78 ± 12.17 years, *P* = 0.003). And these patients showed lower levels of hemoglobin (120.69 ± 24.73 g/l vs. 130.24 ± 21.48 g/l, *P* < 0.001), albumin (24.8 ± 3.98 g/l vs. 38.99 ± 7.98 g/l, *P* < 0.001), eGFR (68.52 ± 33.04 ml/min vs. 93.11 ± 31.08 ml/min, *P* < 0.001) and higher levels of serum creatinine (127.34 ± 114.88umo/l vs. 90.89 ± 55.48 umol/l, *P* < 0.001), serum uric acid (400.52 ± 107.04 umol/l vs. 361.4 ± 106.45 umol/l, *P* = 0.008), urinary red blood cell count [median 84 (13 ~ 205) /ul vs. 12 (5 ~ 34.52) /ul, *P* < 0.001] and urinary protein [median 5160 (4100 ~ 6444) g/24 h vs. 1134 (602 ~ 2106) g/24 h, *P* < 0.001] than patients without NS. As for pathological parameters, more patients with NS showed a higher prevalence of E1 lesions (76.3% vs. 34.3%, *P* < 0.001), T1/2 lesions (86.4% vs. 62.3%, *P* < 0.001) and C1/2 lesions (86.4% vs. 62.68%, *P* < 0.001). There was no significant difference between the two groups in other indexes, including gender, blood pressure, IgA, complement C3, C4, mesangial cell proliferation (M1) and glomerulosclerosis (S1) (Table 1).

**Table 1:** Comparison of baseline characteristics between patients with and without nephrotic syndrome before matching in IgA nephropathy

Variables	NS group (*n* = 59)	non-NS group (*n* = 954)	*P* Value
Age (y)	42.69 ± 14.96	37.78 ± 12.17	0.003
Gender (M/F)	26/33	450/504	0.643
SBP (mm Hg)	124 ± 14	125 ± 13	0.84
HB (g/l)	120.69 ± 24.73	130.24 ± 21.48	< 0.001
Scr (umol/l)	127.34 ± 114.88	90.89 ± 55.48	< 0.001
eGFR (ml/min.1.73m^2^)	68.52 ± 33.04	93.11 ± 31.08	< 0.001
Proteinuria (g/24 h, median,IQR)	5160 (4100 ~ 6444)	1134 (602 ~ 2106)	< 0.001
Uric acid (umol/L)	400.52 ± 107.04	361.40 ± 106.45	0.008
Total IgA (mg/ml)	295.62 ± 111.48	324.71 ± 138.15	0.127
Alb (g/l)	24.8 ± 3.98	38.99 ± 7.98	< 0.001
C3 (mg/dl)	92.88 ± 27.70	95.97 ± 44.20	0.597
C4 (mg/dl)	25.16 ± 8.98	26.58 ± 7.48	0.475
Urine RBC (/ul,median,IQR)	84 (13 ~ 205)	12 (5 ~ 34.52)	< 0.001
M (M0/M1)	5/54	28/926	0.186
E (E0/E1)	14/45	627/327	< 0.001
S (S0/S1)	14/45	337/617	0.293
T (T0/T1/T2)	8/29/22	360/399/195	< 0.001
C (C0/C1/C2)	8/20/31	356/485/113	< 0.001

*SBP* Systolic Pressure, *HB* Hemoglobin, *Scr* Serum creatinine, *eGFR* estimated Glomerular Filtration Rate, *Alb* Albumin, *MEST-C:M* Mesangial hypercellularity, *E* Endocapillary hypercellularity, *S* Segmental glomerulosclerosis, *T* Tubular atrophy/interstitial fibrosis, *C* Crescent

### Clinical and pathological characteristics of patients in IgAN-NS and IgAN-NR groups

Because of the significant differences in baseline characteristics between the patients with and without NS, we selected 57 patients with massive proteinuria and normal serum albumin who were comparable to 59 patients with NS by the propensity score matching method to explore the association between serum albumin with clinical- pathological features and long-term outcome. As shown in Table 2, the median follow-up time was 18.5 (range 12–41.75) months. Patients with NS had lower levels of hemoglobin (120.69 ± 24.73 g/l vs. 134.9 ± 20.7 g/l, *P* = 0.001), albumin (24.8 ± 3.98 g/l vs. 34.82 ± 1.16 g/l, *P* < 0.001) and IgG (653.76 ± 224.83 mg/ml vs. 894.70 ± 223.73 mg/ml, *P* < 0.001) and higher levels of TC (7.46 ± 2.93 mmol/l vs. 5.73 ± 1.54 mmol/l, *P* = 0.02), LDL (4.45 ± 1.86 mmol/l vs. 3.27 ± 1.16 mmol/l, *P* = 0.02), FIB (4.37 ± 1.42 g/l vs. 3.65 ± 0.53 g/l, *P* = 0.003) and D-D (817.94 ± 606.54 ng/ml vs. 373.31 ± 274.8 ng/ml, *P* < 0.001) as well as more severe E1 lesions (76.3% vs. 49.1%, *P* = 0.002) and C1/2 lesions (86.4% vs. 80.7%, *P* = 0.046) than those in NR group. The S1 lesion was more severe in the NR group than that in the NS group (91.2% vs. 76.3%, *P* = 0.03). There was no significant difference between the two groups in gender, age, systolic blood pressure, urinary protein, serum creatinine, eGFR, uric acid, urinary red blood cell count, IgA, complement C3, C4, TG, CRP, mesangial cell proliferation (M1), tubular atrophy/interstitial fibrosis (T1/2), RASI, steroid or immunosuppressive therapy application (Table 2).

**Table 2:** Comparison of baseline characteristics between patients with nephrotic syndrome and nephrotic-range proteinuria after matching in IgA nephropathy

Variables	NS group (*n* = 59)	NR group (*n* = 57)	*P* Value
Age (y)	42.69 ± 14.96	40.67 ± 11.33	0.413
Gender (M/F)	26/33	28/29	0.585
SBP (mm Hg)	124 ± 14	130 ± 23	0.434
HB (g/l)	120.69 ± 24.73	134.9 ± 20.70	0.001
Scr (umol/l)	127.34 ± 114.88	143.5 ± 86.09	0.393
eGFR (ml/min.1.73m^2^)	68.52 ± 33.04	61.58 ± 28.77	0.231
Proteinuria (g/24 h, median, IQR)	5160 (4100 ~ 6444)	5040 (4275 ~ 5770)	0.557
Uric acid (umol/L)	400.52 ± 107.04	438.16 ± 105.07	0.062
Alb (g/l)	24.81 ± 3.98	34.82 ± 1.16	< 0.001
C3 (mg/dl)	92.88 ± 27.70	95.49 ± 15.5	0.555
C4 (mg/dl)	25.16 ± 8.98	25.83 ± 7.88	0.343
Urine RBC (/ul,median,IQR)	84.78 (17.62 ~ 206.12)	94.4 (44.12 ~ 206.12)	0.324
TC (mmol/l)	7.46 ± 2.93	5.73 ± 1.54	0.02
TG (mmol/l)	2.83 ± 1.89	2.74 ± 1.52	0.824
LDL (mmol/l)	4.45 ± 1.86	3.27 ± 1.16	0.02
Total IgA (mg/ml)	295.62 ± 111.48	310.38 ± 124.33	0.536
Total IgG (mg/ml))	653.76 ± 224.83	894.70 ± 223.73	< 0.001
D-dimer (ng/ml)	817.94 ± 606.54	373.31 ± 274.8	< 0.001
FIB (g/l)	4.37 ± 1.42	3.65 ± 0.53	0.003
CRP (mg/dl)	0.54 ± 0.57	0.32 ± 0.23	0.068
Steroid (treatment%)	57 (96.6%)	53 (93%)	0.435
Immunosuppressor (treatment%)	33 (55.9%)	34 (59.6%)	0.685
The RAS inhibitor (treatment%)	31 (52.5%)	27 (47.4%)	0.401
Follow up time (months)	31.53 ± 28.58	29.12 ± 26.66	0.641
Pathological findings			
M (M0/M1)	5/54	2/55	0.262
E (E0/E1)	14/45	29/28	0.002
S (S0/S1)	14/45	5/52	0.03
T (T0/T1/T2)	8/29/22	12/27/18	0.593
C (C0/C1/C2)	8/20/31	11/29/17	0.046

*SBP* Systolic Pressure, *HB* Hemoglobin, *Scr* Serum creatinine, *eGFR* estimated Glomerular Filtration Rate, *Alb* Albumin, *TC* Total Cholesterol, *TG* Triglyceride, *LDL* Low Density Lipoprotein, *MEST-C: M* Mesangial hypercellularity, *E* Endocapillary hypercellularity, *S* Segmental glomerulosclerosis, *T* Tubular atrophy/interstitial fibrosis, *C* Crescent

### Primary outcome analysis

**Fig. 1:**
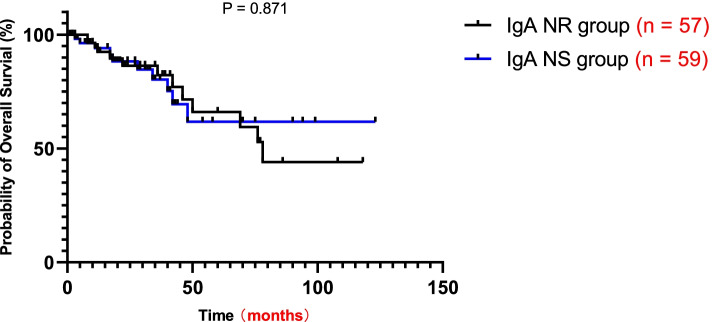
Kaplan–Meier renal survival curves

During the median follow-up of 18.5 months (range 12.0–41.75 months), 13 patients (22%) in the NS group reached the primary outcome compared with 10 (17.5%) in the NR group. As shown by Kaplan–Meier analysis, there was no significant difference in long-term outcome between the two groups (Fig. 1). In addition, using univariate logistic regression, we found that serum albumin level or the presence of hypoalbuminemia was not a risk factor affecting long-term outcome in patients with massive proteinuria (Table 3). After adjusting for eGFR, SBP, uric acid, UTP, T and C, only SBP at baseline (OR = 1.047, 95% CI: 1.009–1.087, *P* = 0.016) was an independent predictor for long-term outcome in patients with massive proteinuria in multivariate analysis (Table 3).

**Table 3:** Logistic regression analysis of factors affecting primary outcome

Variables	Univariate analysis		Multivariate analysis	
	OR (95%CI)	*P*	OR (95%CI)	*P*
Age (y)	1.006 (0.972–1.041)	0.752		
eGFR (ml/min.1.73m^2^)	0.965 (0.947–0.985)	< 0.001	0.980 (0.955–1.005)	0.119
SBP (mm Hg)	1.062 (1.029–1.096)	< 0.001	1.047 (1.009–1.087)	0.016
ALB (g/l)	0.983 (0.913–1.058)	0.647		
ALB high group (versus low group)	0.753 (0.300–1.888)	0.545		
Uric acid (umol/l)	1.004 (1.000–1.009)	0.04	1.001 (0.995–1.007)	0.823
TC (mmol/l)	0.744 (0.511–1.083)	0.12		
TG (mmol/l)	0.742 (0.453–1.213)	0.234		
LDL (mmol/l)	0.819 (0.535–1.254)	0.358		
UTP (g/24 h)	1 (1.000–1.001)	0.03	1 (1.000–1.001)	0.223
M1 (versus M0)	1.517 (0.174–13.263)	0.706		
S1 (versus S0)	0.913 (0.272–3.068)	0.884		
E1 (versus E0)	1.872 (0.676–5.187)	0.228		
T1 + T2 (versusT0)	3.815 (1.668–8.726)	0.002	2.656 (0.869–8.114)	0.087
C1 + C2 (versus C0)	2.395 (1.131–5.073)	0.023	1.898 (0.689–5.230)	0.215

*SBP* Systolic Pressure, *HB* Hemoglobin, *eGFR* estimated Glomerular Filtration Rate, *Alb* Albumin, MEST-C, *M* Mesangial hypercellularity, *E* Endocapillary hypercellularity, *S* Segmental glomerulosclerosis, *T* Tubular atrophy/interstitial fibrosis, *C* Crescent

## Discussion

To date, NS has not been well characterized in patients with IgAN. In this study, we analyzed the baseline data of IgAN patients with and without nephrotic syndrome and found that patients with NS had more severe conditions. Currently, there are limited clinical studies focused on the association between serum albumin and the progression of kidney disease in IgAN patients with massive proteinuria. We compared the data of patients with NS and nephrotic-range proteinuria. Patients with NS had lower levels hemoglobin, albumin and IgG and higher levels of TC, LDL, FIB and D-dimer as well as more severe E1 and C1/2 lesions than those in NR group. While the proportion of segmental glomerulosclerosis in the NR group was higher than that in the NS group. In addition, the prognosis of NS in patients with IgAN was not superior to that of patients with NR. These results suggest that the presence of hypoalbuminemia is only associated with clinical-pathological features, but not associated with progression of IgAN.

Nephrotic syndrome in IgAN was initially thought to represent an associated minimal change type podocyte injury [12]. Subsequent reports indicated the presence of focal segmental sclerosis in IgAN [13–15]. In this study, patients with IgAN superimposed MCD were excluded. Because these patients have a favorable treatment response and outcomes similar to MCD [16, 17]. The proportion of NS presented in IgAN adult patients in our cohort was consistent with previous studies showing NS occurs in fewer than 10% of patients with IgAN [5, 6]. For the clinical and pathological features between NS and non-NS groups in our study, the levels of hemoglobin and albumin were lower in patients with NS. And the levels of serum creatinine, serum uric acid, urinary protein and the prevalence of E1 lesions, T1/2 lesions and C1/2 lesions were higher in NS group. These features suggested IgAN patients with NS had more severe conditions. The significant difference between disease spectrum of NS and non-NS suggested that they may have different pathogenic mechanisms and represent two distinct identifies.

IgAN was one of the most common causes of nephrotic-range proteinuria in kidney disease [12]. Chen et al. showed that normoalbuminemia is associated with IgAN in patients with primary glomerular diseases and nephrotic-range proteinuria [18]. Besides, they observed the higher proportion of IgAN in nephrotic-range proteinuria group compared with NS group (21.4% vs 7.4%). In clinical practice, we observed that some IgAN patients do not develop hypoalbuminemia despite the presence of massive proteinuria. These clinical observations prompted us to review all our patients with IgAN and massive proteinuria to investigate whether the absence or presence of hypoalbuminemia could affect clinical-pathological features and prognosis. After matching gender, age, blood pressure, renal function and proteinuria, the levels of TC, LDL, FIB and D-D were higher in NS group. NS is always associated with systemic complications including coagulopathy, thrombosis and metabolic derangement. Yang et al. pointed out that D-dimer was the most serious risk factor of pulmonary embolism in nephrotic syndrome, which may be used as an indication to prevent anticoagulation [19]. At present, there is still a lack of predictors of thromboembolic events in IgAN complicated with NS in clinical work. Whether the increase of D-dimer can be used as an indicator of thromboembolism and anticoagulant therapy for IgAN with NS needs further research [20]. We also observed patients with NS had more percentages of E1 and C1/2 lesions and a lower percentage of S1. The results suggest that the presence of hypoalbuminemia is associated with acute active lesions in renal pathology. While NR in IgAN reflects coexistent secondary focal segmental glomerular sclerosis or development of extensive glomerulosclerosis and tubulointerstitial fibrosis [14, 21]. As observed previously, nephrotic syndrome in patients with IgAN is always associated active acute lesions in renal pathology. It may be caused by obvious symptom of edema, which urged patients to visit doctors as soon as possible.

The reason why serum albumin level is normal despite the patients had a large amount of proteinuria in NR group is still uncertain. One possible explanation is the change in capillary permeability. Previous studies showed that patients with NS had significantly higher extravasation rate of albumin from vessel compared with healthy controls [22]. This ‘non-renal’ loss of albumin into the interstitium led to the decline of albumin and accumulation of fluid in the body [12].

Besides, our study was one of the few studies concerning the relationship between serum albumin decline and the disease progression in patients with massive proteinuria. Corticosteroids do not consistently result in heavy proteinuria reduction and provide a favorable responsiveness in patients with NS. There was no difference in the use of corticosteroids and immunosuppressive therapy between two groups. The findings show that massive proteinuria in IgAN does not inevitably indicate a poor prognosis. The results supported the notion that the serum albumin was not associated with kidney survival after matching. Consistent with our findings, previous studies demonstrated that serum albumin was not the predictor of renal survival in IgAN [23, 24]. Kawai et al. reported low serum albumin level is an independent risk factor for ESRD in patients with IgAN after adjustment for clinical parameters, including urinary protein excretion, and pathological parameters [25]. While Konieczny et al. revealed serum albumin concentration had a negative influence on the decline in eGFR in IgA nephropathy [26]. Considering the inconsistent results from several studies, a well-designed, randomized, controlled study is required to address this unresolved issue in IgAN with massive proteinuria.

## Conclusion

In conclusion, a prevalence of 5.8% of NS was presented in IgAN adult patients in our study. IgAN with NS patients had low levels of hemoglobin, albumin, high levels of serum creatinine, serum uric acid, urinary protein and more acute lesions. The prognosis of NS in patients with IgAN was not inferior to that of patients with nephrotic range proteinuria and normal serum albumin.

## Data Availability

Raw data used during the current study are available from the corresponding author on reasonable request for non-commercial use.

## References

[1] Rajasekaran A, Julian BA, Rizk DV (2021). IgA nephropathy: an interesting autoimmune kidney disease. Am J Med Sci.

[2] Suzuki H, Kiryluk K, Novak J, Moldoveanu Z, Herr AB, Renfrow MB, Wyatt RJ, Scolari F, Mestecky J, Gharavi AG, Julian BA (2011). The pathophysiology of IgA nephropathy. J Am Soc Nephrol.

[3] Li X, Liu Y, Lv J, Shi S, Liu L, Chen Y, Zhang H (2014). Progression of IgA nephropathy under current therapy regimen in a Chinese population. Clin J Am Soc Nephrol.

[4] Moriyama T (2019). Clinical and histological features and therapeutic strategies for IgA nephropathy. Clin Exp Nephrol.

[5] Han X, Xiao Y, Tang Y, Zheng X, Anwar M, Qin W (2019). Clinical and pathological features of immunoglobulin a nephropathy patients with nephrotic syndrome. Clin Exp Med.

[6] Kim JK, Kim JH, Lee SC, Kang EW, Chang TI, Moon SJ, Yoon SY, Yoo TH, Kang SW, Choi KH, Han DS, Kie JH, Lim BJ, Jeong HJ, Han SH (2012). Clinical features and outcomes of IgA nephropathy with nephrotic syndrome. Clin J Am Soc Nephrol.

[7] Alshomar AA (2016). Nephrotic syndrome is a rare manifestation of IGA nephropathy. Int J Health Sci (Qassim).

[8] Chi CJ, Chen YC, Chen HH, Yeh JC (2000). Pathological differences in nephrotic-range proteinuria with and without hypoalbuminemia. Nephron.

[9] Praga M, Borstein B, Andres A, Arenas J, Oliet A, Montoyo C, Ruilope LM, Rodicio JL (1991). Nephrotic proteinuria without hypoalbuminemia: clinical characteristics and response to angiotensin-converting enzyme inhibition. Am J Kidney Dis.

[10] Wang J, Xie P, Huang JM, Qu Y, Zhang F, Wei LG, Fu P, Huang XJ (2016). The new Asian modified CKD-EPI equation leads to more accurate GFR estimation in Chinese patients with CKD. Int Urol Nephrol.

[11] Trimarchi H, Barratt J, Cattran DC, Cook HT, Coppo R, Haas M, Liu ZH, Roberts IS, Yuzawa Y, Zhang H, Feehally J (2017). Oxford classification of IgA nephropathy 2016: an update from the IgA nephropathy classification working group. Kidney Int.

[12] Ng JK, Ma TK, Lai FM, Chow KM, Kwan BC, Leung CB, Li PK, Szeto CC (2018). Causes of nephrotic syndrome and nephrotic-range proteinuria are different in adult Chinese patients: a single centre study over 33 years. Nephrology (Carlton).

[13] Ulusoy S, Ozkan G, Sonmez M, Mungan S, Kaynar K, Cansiz M, Kazaz N (2010). Absence of hypoalbuminemia despite nephrotic proteinuria in focal segmental glomerulosclerosis secondary to polycythemia vera. Intern Med.

[14] KDIGO (2021). clinical practice guideline for the management of glomerular diseases. Kidney Int.

[15] Packham DK, Yan HD, Hewitson TD, Nicholls KM, Fairley KF, Kincaid-Smith P, Becker GJ (1996). The significance of focal and segmental hyalinosis and sclerosis (FSHS) and nephrotic range proteinuria in IgA nephropathy. Clin Nephrol.

[16] Herlitz LC, Bomback AS, Stokes MB, Radhakrishnan J, D'Agati VD, Markowitz GS (2014). IgA nephropathy with minimal change disease. Clin J Am Soc Nephrol.

[17] Li XW, Liang SS, Le WB, Cheng SQ, Zeng CH, Wang JQ, Liu ZH (2016). Long-term outcome of IgA nephropathy with minimal change disease: a comparison between patients with and without minimal change disease. J Nephrol.

[18] Chen M, Zhou FD, Zhao MH, Wang HY (2011). Normoalbuminaemia is associated with IgA nephropathy in primary glomerulopathy with nephrotic-range proteinuria in Chinese patients. Nephrol Dial Transplant.

[19] Yang Y, Lv J, Zhou F, Chen M, Wang R, Zhao M, Wang H (2014). Risk factors of pulmonary thrombosis/embolism in nephrotic syndrome. Am J Med Sci.

[20] Pattrapornpisut P, Avila-Casado C, Reich HN (2021). IgA nephropathy: core curriculum 2021. Am J Kidney Dis.

[21] Matsuzawa N, Nakabayashi K, Nagasawa T, Nakamoto Y (2002). Nephrotic IgA nephropathy associated with disseminated tuberculosis. Clin Nephrol.

[22] Rostoker G, Behar A, Lagrue G (2000). Vascular hyperpermeability in nephrotic edema. Nephron.

[23] Stefan G, Stancu S, Boitan B, Zugravu A, Petre N, Mircescu G (2020). Is there a role for IgA/C3 ratio in IgA nephropathy prognosis? An outcome analysis on an european population. Iran J Kidney Dis.

[24] Caliskan Y, Ozluk Y, Celik D, Oztop N, Aksoy A, Ucar AS, Yazici H, Kilicaslan I, Sever MS (2016). The clinical significance of uric acid and complement activation in the progression of IgA nephropathy. Kidney Blood Press Res.

[25] Kawai Y, Masutani K, Torisu K, Katafuchi R, Tanaka S, Tsuchimoto A, Mitsuiki K, Tsuruya K, Kitazono T (2018). Association between serum albumin level and incidence of end-stage renal disease in patients with Immunoglobulin a nephropathy: a possible role of albumin as an antioxidant agent. PLoS One.

[26] Konieczny A, Donizy P, Golebiowski T, Tukiendorf A, Halon A, Kusztal M, Augustyniak-Bartosik H, Krajewska M (2021). Clinical and histopathological factors influencing IgA nephropathy outcome. Diagnostics (Basel).

